# Characteristics of Asthma Exacerbations in Emergency Care in Switzerland—Demographics, Treatment, and Burden of Disease in Patients with Asthma Exacerbations Presenting to an Emergency Department in Switzerland (CARE-S)

**DOI:** 10.3390/jcm12082857

**Published:** 2023-04-13

**Authors:** Marco Rueegg, Jeannette-Marie Busch, Peter van Iperen, Joerg D. Leuppi, Roland Bingisser

**Affiliations:** 1 Emergency Department, University Hospital Basel, 4031 Basel, Switzerland; 2 University Center of Internal Medicine, Cantonal Hospital Baselland, 4410 Liestal, Switzerland; 3 Department of Respiratory and Immunology Biologics, AstraZeneca AG, 6340 Baar, Switzerland; 4 Medical Faculty, University of Basel, 4056 Basel, Switzerland

**Keywords:** asthma exacerbation, emergency care, asthma treatment pattern, GINA 2021, medical shortcomings

## Abstract

Emergency care for asthma is provided by general practitioners, pulmonologists, and emergency departments (EDs). Although it is known that patients presenting to EDs with acute asthma exacerbations are a vulnerable population and that this mode of presentation is a risk marker for more severe complications, research on this population is scarce. We conducted a retrospective study on patients with asthma exacerbations who presented to the ED of the University Hospital Basel, Switzerland, during 2017–2020. Of the last 200 presentations, 100 were selected and analyzed to assess demographic information, the use of previous and ED-prescribed asthma medication, and clinical outcomes after a mean period of time of 18 months. Of these 100 asthma patients, 96 were self-presenters, and 43 had the second highest degree of acuity (emergency severity index 2). Global Initiative for Asthma (GINA) step 1 and step 3 were the most common among patients with known GINA levels, accounting for 22 and 18 patients, respectively. A total of 4 patients were undergoing treatment with oral corticosteroids at presentation, and 34 were at discharge. At presentation, 38 patients used the combination therapy of inhaled corticosteroid/long-acting β_2_-agonist (ICS/LABA), and 6 patients underwent ICS monotherapy. At discharge, 68 patients were prescribed with ICS/LABA. At entry to the ED, about one-third of patients did not use any asthma medication. In total, 10 patients were hospitalized. None of them needed invasive or non-invasive ventilation. A follow-up for the study was precluded by the majority of patients. This group of asthma patients seemed particularly vulnerable as their asthma medication at presentation was often not according to guidelines or even lacking, and almost all the patients had self-presented to the ED without any reference from a physician. The majority of patients did not give consent to the collection of any follow-up information. These medical shortcomings reflect an urgent medical need to improve care for patients at high risk of asthma exacerbations.

## 1. Introduction

Asthma is the most common chronic non-infectious disease, affecting over 300 million people globally [1]. It causes respiratory symptoms including coughing, chest tightness and shortness of breath, and is associated with variable expiratory airflow limitation and with airway inflammation [2]. Typically, patients with asthma report periods of worsening symptoms and worsening airway obstruction, called exacerbations, that can at times be fatal. Asthma exacerbations are defined as imbalances in the asthmatic disorder and are provoked by external agents (e.g., viral infection, allergen exposure, air pollution), poor compliance with treatment, or both. In general, exacerbations are severe complications in asthma patients. Exacerbations are often unpredictable and can affect all severities of the disease. Severe exacerbations can even occur in patients with mild asthma. Patients with infrequent asthma symptoms can still experience severe or fatal exacerbations [3]. Poor symptom control, however, is burdensome for patients and increases the risk of exacerbations. In order to reduce the risk of exacerbations and develop better symptom control, all patients with asthma should receive ICS-containing controller treatment independently of age according to Global Initiative for Asthma (GINA) [2]. For safety reasons, GINA no longer recommends the treatment of asthma with short-acting β_2_-agonists (SABA) alone in those patients. According to these international guidelines, all patients with asthma should be provided with guided self-management education including symptom monitoring and/or lung function monitoring, a written personal action plan for management of asthma, and frequent reviews by a physician [2].

The Royal College of Emergency Medicine (RCEM) stated in its last clinical audit on asthma management in the UK [4,5] that most emergency departments (EDs) do not reach the standard level of care for patients presenting with asthma exacerbation. Compared to similar antecedent audits from the years 2009/2010 and 2013/2014, a decline in performance was evident [6]. Swiss Guidelines for the management of asthma exacerbations based on GINA exist and have been updated [7]. Due to the absence of routine assessment of patients with asthma exacerbation, there is a lack of recent data in Switzerland concerning its management [8,9].

As multiple international audits have indicated a considerable need for improvement in order for existing guidelines to be met [10,11,12,13,14,15], we decided to further analyze Switzerland’s situation. We therefore aimed to assess the management of asthma exacerbations at the University Hospital Basel in Switzerland between 2017 and 2020, placing a special focus on previous asthma treatment and asthma severity as well as on the treatment at discharge in order to explore clinical realities concerning exacerbations and possible shortcomings in terms of asthma treatment.

## 2. Methods

### 2.1. Study Design

This non-interventional, observational, and retrospective chart review (single-center, single arm) was conducted at University Hospital Basel, Switzerland, to assess demographic characteristics, treatment, and burden of disease in patients with asthma presenting to the ED due to an exacerbation. Data were collected in 2020 to comprise a retrospective consecutive sample from 2017 to 2020. The hospital database of patients admitted to the ED for asthma exacerbations was screened to assess 200 asthma exacerbations during a period of 36 months prior to a defined index date. Based on both eligibility criteria and the availability of current patient health data, 50% of patients out of these 200 identified asthma exacerbation patients qualified for inclusion, leading to an anticipated convenience sample of 100 patients for use in the chart review. For each patient, only the last exacerbation was collected in the electronic case report form (eCRF). Follow-up data were assessed via physicians treating asthma or the patient themselves. It was also recorded if patients or physicians could not be contacted. 

### 2.2. Eligibility Criteria

Patients (≥18 years) with a primary diagnosis of asthma according to the guidelines of the American Thoracic Society and the European Respiratory Society (ATS/ERS) [16], after confirmation by a physician, were eligible for the study, if they had been admitted one or several times to the ED due to asthma exacerbation during the 36 months prior to the index date. Written informed consent was needed from the patients for the collection of follow-up data after discharge from the ED. Patients were excluded if they had been admitted to the ED due to chronic obstructive pulmonary disease (COPD). Additionally, patients who refused consent to the use of their data were excluded.

### 2.3. ED Process and Measurements

According to standard procedure, each patient referred by general practitioner (GP), specialist, emergency medical service (EMS), or self-referred to the ED was evaluated at triage by a triage nurse. There, along with demographics and vital signs, the emergency severity index (ESI) was assessed, this being a five-level triage index scale: the highest acuity level, ESI 1, is defined as “in need of life-saving intervention”; ESI 2 is defined as “acute emergency if not seen by a physician within 10 min”; ESI 3 is defined as “in need of more than two external resources”; ESI 4 is defined as “in need of one external resource”, and ESI 5 is defined as “in need of no external resource” [17,18]. Patients were then assessed by a physician, who inquired about medical history, current medication, and comorbidities. For this study, specific comorbidities defined as “comorbidities of interest” were nasal polyposis, chronic rhinosinusitis, gastroesophageal reflux disease (GERD), depression/anxiety, obstructive sleep apnoea (OSA), cardiovascular diseases, metabolic diseases, atopic dermatitis, and aspirin-exacerbated respiratory disease (AERD). At admission, patients were characterized according to their asthma GINA step along the five-level GINA scale. This was done based on their controller medication [2]: GINA steps 1 and 2 (low-dose inhaled corticosteroid (ICS)/formoterol on demand), GINA step 3 (low-dose inhaled corticosteroid (ICS)/formoterol maintenance), GINA step 4 (medium-dose inhaled corticosteroid (ICS)/formoterol maintenance), and GINA step 5 (medium-/high- dose ICS/formoterol maintenance, add-on LAMA, and biologicals). Once an asthma exacerbation had been diagnosed, the exacerbation severity was defined by considering the patients’ clinical findings. An adequate treatment comprising of oxygen, corticosteroids, antihistamines (e.g., cetiricin, clemastine), and other potentially asthma-related medication was administered. In case of a severe, potentially life-threatening exacerbation, patients were intubated. Pulmonologists were consulted when needed. Possible adverse events associated with the medication administered were recorded and treated appropriately. The current asthma medication was reevaluated and, if necessary, an escalated asthma medication on a higher GINA step was prescribed. If needed, a laboratory examination was performed consisting of blood–gas analysis as well as an eosinophil count. Following a diagnostic work-up and acute treatment, the patients’ care path (out-hospital treatment, hospitalization, admission to intensive care unit (ICU)) were defined. 

### 2.4. Follow-Up

Asthma treatment specialists (pulmonologists, allergists) and/or general practitioners of patients who had provided written informed consent were contacted by mail or email 6 to 31 months (mean 18 months) after their discharge from hospital. Patients who were not undergoing any kind of ambulatory treatment of asthma were asked to provide the address of the last health care facility (hospital, rehabilitation center) they were treated at. Practitioners were asked to either provide information or send a current medical examination report regarding the patients’ asthma status. Data of interest for the follow-up were the course of disease (number of further asthma exacerbations), laboratory results including eosinophil count, and information on the current GINA step and the patients’ adherence to prescribed medication. 

### 2.5. Study Objectives and Statistics

The primary endpoint of the study was to assess the number and severity of asthma exacerbations according to the ESI level documented in the medical records of patients admitted to the ED during the study period. The secondary objective was the characterization of asthma treatment (medication at admission and discharge) and outcomes in the ED. As an exploratory endpoint, the current status of patients after ED-managed exacerbations was assessed in the follow-up.

Continuous variables were summarized using descriptive statistics including total observations equal to total number of records per case (*n*), arithmetic mean, standard deviation (SD), median, minimum, maximum, 1st and 3rd quartile. Categorical data were summarized by the number and percentage of patients or entries in each category.

2KMM (Katowice, Poland) provided the software for data collection by eCRF (GoResearch^TM^) and performed all statistical analyses, everything in compliance with good clinical practice (GCP) requirements.

### 2.6. Ethics

The responsible ethics commission of Nordwest- and Zentralschweiz approved the study (BASEC number 2020-01486). It was conducted in accordance with the protocol, the Declaration of Helsinki, the principles of good clinical practice, the Human Research Act (HRA), and the Human Research Ordinance (HRO), as well as locally relevant regulations.

## 3. Results

### 3.1. Patient Demographics and Clinical Assessments at Admission

For this retrospective chart review, 200 asthma exacerbation cases were identified as having been treated in the ED due to asthma exacerbation during the study period. Based on the eligibility criteria, 100 patients were enrolled in the study (Table 1). At admission, patients’ mean age was 35.9 years (SD 13.5 y) in a range of 16–82 years. Patients’ age at initial diagnosis of asthma was available for a quarter of patients (26%), with a mean age of 29.0 years (range of 2–80 y). Most patients (96%) were admitted without a reference from any kind of physician, i.e., they self-presented, or were referred by an EMS. In total, two patients each were referred from pulmonologists and GPs. 

According to the ESI, the majority of patients (97.0%) were classified as being at levels 2–4 of the 5-level triage index scale at admission. The second highest acuity degree, ESI level 2, was the most common and was assigned to 43.0% of all patients. GINA step 1 and step 3 were the most common, constituting 22.0% and 18.0%, respectively, of all patients. For 50% of patients, however, information about GINA step at admission was not available (Table 2).

### 3.2. Asthma Treatments at Admission and Discharge

At admission to the ED, the patients’ current asthma medication was recorded. The two most frequently prescribed medication classes were SABA (57 entries) and the combination therapy of ICS/LABA (38 entries). ICS monotherapy was prescribed for 6 patients (Table 3). Oral corticosteroids (OCS) were prescribed for 2 patients using prednisone (15 mg daily) and prednisolone (dosage unknown), although OCS use was unknown for 2 patients. In total, 96% of patients did not use any OCS. The use of biological treatment was not confirmed in any case. About one-third of patients (30%) did not use any asthma ‘controller or reliever’ (inhaled medication only except leukotriene receptor antagonist (LTRA) for oral intake) consisting of ICS, LABA, LAMA, SABA, short-acting muscarinic antagonists (SAMA), LTRA or their combinations. 

Compared to the status at admission, SABA (71 entries) and ICS/LABA (68 entries) were more frequently prescribed at discharge. OCS treatment was prescribed for 34 patients, with 4 patients using prednisolone and 30 patients using prednisone. A median daily dosage of 40 mg OCS (prednisone equivalent) was used for a duration of 3–5 days (mean duration 4.7 days) before discontinuation or dose reduction. Biological treatment was not prescribed to any patient. ‘Controller and reliever’ were recorded for 91% of all patients, and only 5% of patients were not prescribed any of these drugs at discharge from the ED.

In summary, compared to admission, an increased number of patients received OCS (4 patients at admission versus 34 patients at discharge) as well as ICS/LABA (38 patients at admission versus 68 patients at discharge). The proportion of the remaining classes of medications did not change significantly between admission and discharge.

### 3.3. Management of Asthma Exacerbations in ED and Duration of Stay

At ED presentation due to asthma exacerbation, several parameters were evaluated for detailed clinical diagnosis. In more than half of the patients, venous blood gas analyses (*n* = 53) and eosinophil counts from peripheral blood (*n* = 66) were performed. The mean pH was 7.42 (SD 0.05), mean PaCO_2_ 5.37 kPa (SD 1.04), mean HCO_3_ 24.90 mmol/L (SD 2.75), and mean eosinophil count was 0.38 G/L (SD 0.33).

During the ED work-up process, 39% of patients received corticosteroids consisting both of OCS as well as parenteral corticosteroids, with dosage ranging from 40 mg once daily up to 125 mg twice daily. Parenteral corticosteroids were administered almost twice as often as OCS (27 entries vs. 15 entries). Asthma ‘controller and reliever’ medications were recorded for 71% of all patients but were lacking in 26% of patients. Additionally, some patients were treated with other asthma exacerbation-related medications such as antibiotics (3%) and antihistamines (8%), and other medications like adrenaline, magnesium, and painkillers (11%). In 5 cases, oxygen was administered. The use of biologics was not confirmed during the ED work-up.

Among all patients presenting to the ED, 10% were hospitalized. None of them needed invasive or non-invasive ventilation. Additionally, no patient was admitted to an ICU. The mean hospital stay was one day. One female patient remained in hospital for 15 days. 

At discharge from the ED, patients were mainly referred to pulmonologists (24%) or GPs (15%). Several patients were referred to allergists (3%) and one patient was referred to a rehabilitation center. In most cases, however, patients were neither referred to specialists or GPs (57%). While only 4% (Table 1) were referred by any physician at presentation, 43% (Table 4) were referred to an asthma treatment physician at discharge.

### 3.4. Clinical Follow-Up

The majority of patients (74%) declined to provide any information about their current health status, and 26% of all patients consented to a follow-up at a mean time point of 18 months after discharge. Most of these patients were currently in treatment at their GPs (*n* = 14) or pulmonologists (*n* = 10). Since their discharge from the ED, 23% of patients (2 male, 4 female) experienced another confirmed asthma exacerbation. One female patient experienced two confirmed asthma exacerbations.

## 4. Discussion

In this retrospective chart review, we analyzed the consecutive data of 100 patients presenting to the ED with asthma exacerbations. The vast majority of asthma exacerbations were of high acuity, with an assigned ESI level of 3 or lower (i.e., higher acuity). Almost half the patients were assigned an ESI level 2. In contrast to the high acuity of exacerbations, almost every patient presented to the ED without reference of any physician (i.e., self-presentation or referral by an EMS). This may also contribute to the fact that no GINA classification was available for half the patients at admission, while a low GINA step (1 to 3) was determined for the other half. Furthermore, insufficient treatment care was obvious in respect to asthma medications at admission. One-third of patients were under no type of treatment with ICS, LABA, LAMA, SABA, or SAMA as mono- or combination therapy. Anti-inflammatory treatment with ICS/LABA was prescribed for 38 patients, and ICS monotherapy was recommended for 6 patients.

Compared to admission, pharmacological treatment was escalated at discharge from the ED, with nine out of ten patients now receiving asthma ‘controller and/or reliever’ medications. SABA use in asthma patients was already highly prevalent at admission. At discharge, the number of SABA prescriptions recorded was even higher, although prescriptions of the ICS/LABA combination only doubled, only covering the treatment of two-thirds of all patients at discharge. However, according to GINA [2], ICS-containing controller therapies should be prescribed in order to reduce the risk of any further exacerbations. Thus, already in GINA step 1, the use of SABA without any concomitant ICS as a controller is no longer recommended because of its lack of efficacy and due to safety reasons [2]. Our findings are in line with the data of a recent asthma audit in a Swiss general hospital that uncovered a discrepancy between GINA guidelines and the actual clinical approaches used: 64% of patients left the ED with reliever medication after asthma exacerbation treatment, whereas only 55% received a new or increased controller therapy with ICS at discharge [9]. We hypothesize that physician factors explaining this discrepancy include reservations towards ICS, the attitude that proper treatment should be prescribed and explained at a follow-up by family physicians or specialists, and the lack of time and knowledge. Patient factors may include a certain fear of suffering side-effects from ICS, concerns about cost of therapy, or side-effects experienced in the past.

In this context, it seems important to note the high relapse rate of around 20% seen in our patients after discharge from the ED, with 6 out of 26 reporting another asthma exacerbation within a mean time frame of 18 months. Recurrent exacerbations are associated with uncontrolled asthma and vice versa [19,20]. A systematic review reported that a median of 17% of patients suffer a relapse within the first 4 weeks after discharge from the ED [21]. In this review, female sex, past healthcare usage, and ICS use at presentation were commonly and significantly associated factors with relapse occurrence. Interestingly, in our study, 4 out of 6 patients reporting another exacerbation in the follow-up period were female asthma patients, with one female even reporting two exacerbations. 

As asthma is a chronic disease and exacerbations may be very acute and severe without the typical and obvious predictors, such as precedent need for ventilatory therapy or hospitalization, every patient presenting to EDs with asthma exacerbation should be evaluated thoroughly, treated in conformity with the guidelines, and referred to an experienced physician for follow-up care. Therefore, GINA recommends collecting comprehensive patient information on the disease, the continuous monitoring of the patient’s symptoms and risk factors by the physician and the patient themselves, issuing a written personal asthma action plan to the patient, educating patients on correct inhaler techniques, and performing regular checks of treatment adherence [2]. In contrast to these guideline recommendations, our data reflect a different clinical reality, with a vast majority of patients self-presenting to the ED and roughly three-quarters of patients unwilling to provide informed consent to inquire further about their current health status. 

The ED plays a critical role in providing acute treatment of asthma exacerbations, but it also has a certain responsibility in our health care system with regard to the treatment of patients with chronic diseases. In the case of asthma, it has a preventive role, with a unique ability to improve the quality of asthma care [22]. Several publications highlight the importance of quality emergency care consisting of appropriate asthma education regarding warning signs, medical management, follow-up recommendations, and reasons for return to the ED [22,23,24]. Compared to usual care (defined as discharge instructions and prescriptions for medications), educational interventions were found to have improved treatment effectiveness to a large extent in a recent review [23]. In a randomized study, Gregoriano et al. showed that closer supervision and instructions of patients can improve adherence and reduce exacerbation rates [25,26]. In a randomized controlled trial performed in the ED, the effectiveness of a patient-centered education (PCE) was compared to that of a standard asthma patient education on ED re-attendance [27]. The authors found that a learner-centered approach to asthma education reduced re-attendance to EDs and, therefore, offered promise as a brief education process in the ED. Furthermore, ED physicians have an exceptional position regarding the referral of patients to GPs or specialists. We believe that part of their duty is to ensure continuous care regardless of acuity at ED presentation. Ideally, EDs, specialists, as well as GPs should communicate in a closed loop, providing follow-up and continuous care. 

In addition to the burden of chronic disease for asthma patients, recurring exacerbations also impose a high burden on society due to losses in productivity and the increase in the use of healthcare resources and their costs [28,29]. The average costs for the management of an adult asthma patient with an acute episode in the ED vary between EUR 330.39 and EUR 808.25 depending on severity, as shown in an Italian study [30]. This finding reflects that discharge information, an inexpensive preventive measure, may be cost-effective even in the ED. Although EDs are often crowded, even in a country with a high physician ratio, such as Switzerland, the need for structured discharge information and physician-to-physician handover is obvious.

The strength of the presented data lies in the availability of data on treatment regimens before and after ED presentation in an environment where standardized evaluation and treatment is highly valued, as well as in the broad distribution of patient characteristics, e.g., age and severity.

There was a possible selection bias, as only half of the 200 identified patients presenting to the ED with asthma exacerbation qualified in terms of eligibility criteria to participate in the study and data availability. However, a good portion of the identified patients was excluded due to indication of concomitance of COPD, which was not in the scope of this study. Furthermore, although the study was not limited to patients with improper treatment, the observation showed that asthma patients presenting to the ED were mostly not properly treated and followed up. Another weakness of our study lay in the incomplete documentation of lung function parameters and follow-up data. One could also argue that the cohort studied was not of very high acuity and severity, as indicated by vital data (e.g., blood gas values) and the 90% discharge rate. However, three-quarters of all patients did not agree to have their follow-up data collected, pointing to a possible feature of this group of asthma patients. This group seemed to be characterized by a lack of care and supervision by GPs and pulmonologists, both before and after ED presentation for exacerbation. Due to the secondary nature of our data in general, data gaps in medical records and unavailability of data need to be acknowledged. Furthermore, a certain bias can be expected due to the loose definition of the term “asthma exacerbation”, even in a specialized environment such as the ED [31].

The future of research on patients presenting to EDs with asthma exacerbations should include evaluations of these patients’ needs and fears, their health literacy, and their understanding of their specific condition. Prospective studies are needed, as this explorative study clearly shows the need to continuously monitor admission and discharge parameters in order to improve the implementation of guidelines that are very inconsistently followed by both patients and caregivers [15,32]. The vast majority of publications in this field consist of guidelines. However, implementation science has not had a huge impact on the real-life practice and treatment of this chronic disease in this vulnerable population of those presenting to EDs in cases of exacerbation, and there is a certain lack of care shown by specialists between exacerbations. The importance of post-discharge care for patients with asthma exacerbations was emphasized in a recent publication [24]. Despite advances in research on asthma, there remain many evidence gaps in managing ED patients with asthma exacerbation according to the authors.

## 5. Conclusions

The main results of this study were the low adherence to current treatment guidelines, both at presentation and after discharge from the ED, the relatively benign course in spite of the high number of patients presenting with high acuity, and the unwillingness of the majority of patients to consent to participate in a study follow-up. In particular, the lack of appropriate anti-inflammatory treatment with ICS, as would be in line with GINA guidelines from step 1 onwards, reflects shortcomings in current asthma treatment and identifies a medical need to improve asthma care for this vulnerable patient population. Although standardized medical protocols for asthma treatment are easily accessible in Switzerland [7,33], there seems to be an obvious gap between adherence by physicians in clinical reality.

## Figures and Tables

**Table 1:** Patient demographics and referral status at admission.

	All (*n* = 100)	Female (*n* = 61)	Male (*n* = 39)
Age (years, mean)	35.9	35.2	36.9
Referred from, *n* (%)			
No physician	96 (96.0%)	57 (93.4%)	39 (100%)
GP	2 (2.0%)	2 (3.3%)	0
Pulmonologist	2 (2.0%)	2 (3.3%)	0

GP, general practitioner. Referral from ‘no physician’ includes self-presentation and referral by EMS.

**Table 2:** Clinical assessment at admission.

	All (*n* = 100)	Female (*n* = 61)	Male (*n* = 39)
ESI level, *n* (%)			
1	1 (1.0%)	1 (1.6%)	0
2	43 (43.0%)	32 (52.5%)	11 (28.2%)
3	32 (32.0%)	20 (32.8%)	12 (30.8%)
4	22 (22.0%)	7 (11.5%)	15 (38.5%)
5	2 (2.0%)	1 (1.6%)	1 (2.6%)
GINA step, *n* (%)			
1	22 (22.0%)	12 (25.6%)	10 (25.6%)
2	5 (5.0%)	4 (2.6%)	1 (2.6%)
3	18 (18.0%)	13 (21.3%)	5 (12.8%)
4	4 (4.0%)	2 (3.3%)	2 (5.1%)
5	1 (1.0%)	1 (1.6%)	0
N/A	50 (50.0%)	29 (47.5%)	21 (53.8%)

ESI, emergency severity index; GINA, Global Initiative for Asthma; N/A, not available. At admission, only several patients had comorbidities of interest. Chronic rhinosinusitis was present in 12% of all patients, this being the most common diagnosis, followed by diagnoses of cardiovascular diseases (8%), and depression/anxiety (8%). The prevalence at admission of nasal polyposis, GERD, metabolic diseases, and atopic dermatitis was ≤4% of all patients, with no confirmed case of OSA or AERD.

**Table 3:** Asthma medications prescribed at admission and at discharge.

Prescribed Medications *n*, (%)	Admission to ED (*n* = 107)	Discharge from ED (*n* = 156)
ICS/LABA	38 (35.5)	68 (43.6)
ICS	6 (5.6)	5 (3.2)
LABA	1 (0.9)	1 (0.6)
LAMA	3 (2.8)	4 (2.6)
LABA/LAMA	1 (0.9)	2 (1.3)
SABA	57 (53.3)	71 (45.5)
SABA/SAMA	1 (0.9)	4 (2.6)
LTRA	0	1 (0.6)

ICS, inhaled corticosteroid; LABA, long-acting β_2_-agonist; LAMA, long-acting muscarinic antagonists; SABA, long-acting β_2_-agonist; SAMA; short-acting muscarinic antagonists; LTRA, leukotriene receptor antagonist.

**Table 4:** Referral at discharge from ED.

Referral to *n*, (%)	All (*n* = 100)	Female (*n* = 61)	Male (*n* = 39)
None	57 (57.0%)	34 (55.7%)	23 (59.0%)
Allergist	3 (3.0%)	2 (3.3%)	1 (2.6%)
GP	15 (15.0%)	7 (11.5%)	8 (20.5%)
Pulmonologist	24 (24.0%)	17 (27.9%)	7 (17.9%)
Rehabilitation center	1 (1.0%)	1 (1.6%)	0

GP, general practitioner.

## Data Availability

The data underlying this study may be shared on reasonable request to the corresponding author and after individual approval of the responsible ethics committee.
